# Risk of Childhood Cancer in Children with Congenital Anomalies and Their Impact on Survival: A Population-Based Registry Approach

**DOI:** 10.3390/curroncol32110621

**Published:** 2025-11-06

**Authors:** Carmen Martos, Laura García-Villodre, Laia Barrachina-Bonet, Lucía Páramo-Rodríguez, Berta Arribas-Díaz, Anna Torró-Gómez, Noura Jeghalef El Karoui, Consol Sabater, Clara Cavero-Carbonell

**Affiliations:** 1 Rare Diseases Research Unit, Foundation for the Promotion of Health and Biomedical Research of the Valencian Region (Fisabio), 46020 Valencia, Spain; 2 Childhood and Adolescent Cancer Population-Based Registry of the Valencian Region, Public Health Directorate, Generalitat Valenciana, 46020 Valencia, Spain

**Keywords:** childhood cancers, congenital anomalies, population-based registries, childhood cancer risk, survival

## Abstract

An increased cancer risk in children with congenital anomalies was reported. This study explored the cancer risk in children aged 0–14 diagnosed with major congenital anomalies during the first year of life, born in 2007–2020 and residing in the Valencian Region, as well as their impact on survival. The Childhood and Adolescent population-based Cancer Registry provided the cancers diagnosed in the period 2007–2020 in children born between 2007 and 2020. They were linked with data from the Congenital Anomalies population-based Registry to identify children with congenital anomalies. Standardized incidence ratio (SIR) and 95% confidence intervals (95% CI) were computed. Kaplan–Meier analysis was used to estimate survival function. Children with congenital anomalies had 5-fold cancer risk compared with the reference population (SIR = 5.5; 95% CI: 4.3–7.0). The highest risk was found for children with genetic disorder anomalies (SIR = 26.6; 95% CI: 17.3–37.8) and children with Down syndrome (SIR = 22.2; 95% CI: 10.1–39.1). Children with cancers and congenital anomalies had worse 5-year survival compared with children having only one of these diseases. These results point out the need to clinical follow-up after congenital anomaly diagnosis. The low number of cases, due to rarity of the childhood cancers and congenital anomalies, was the main limitation of performing more detailed analyses.

## Simple Summary

Childhood cancers and congenital anomalies are rare diseases, but a major cause of death among children. An increased risk of developing cancer in children with congenital anomalies was reported. We evaluated this risk and their impact on survival. The data were obtained from two population-based registries established in the Valencian Region: the Childhood and Adolescent Cancer Registry and the Congenital Anomalies Registry. The study population was children with major congenital anomalies and/or childhood cancers, born between 2007 and 2020. Our study confirmed an increased cancer risk 5-fold in children with congenital anomalies compared with children without this pathology. The highest risk was observed for children with genetic disorder anomalies and children with Down syndrome. In addition, children having childhood cancers and congenital anomalies had worse survival compared with children with only one of these diseases. These results pointed out the need for clinical follow-up after congenital anomaly diagnosis.

## 1. Introduction

Despite advances in treatment, childhood cancer survivors continue to experience substantial comorbidities stemming from chronic health conditions and face an elevated risk of premature mortality compared to the general population [1,2].

Robust evidence supports a consistent association between childhood cancers and congenital anomalies [3,4]. However, the etiology of both conditions remains largely unknown [5,6]. A deeper understanding of the relationship between childhood cancers and congenital anomalies may help identify underlying genetic and/or environmental factors contributing to both pathologies [7,8,9,10,11,12].

Several epidemiological studies have analyzed the overall association between childhood cancer and any congenital anomaly [13,14,15,16,17,18,19,20,21,22,23,24,25,26]. Other researchers have focused on specific childhood cancers, such as neuroblastoma [27,28,29,30], brain and central nervous system tumors [31,32], germ cell tumors [33,34], Langerhans cell histiocytosis [35], and carcinomas [36]. Although an increased risk of childhood cancer has been observed in children with non-chromosomal anomalies [37], this risk is generally lower than that observed in children with chromosomal anomalies [16,20]. Moreover, associations between specific congenital anomalies and particular childhood cancers have been identified—for example, a higher risk of leukemia has been consistently reported in children with Down syndrome [14,15,21,22,38].

Overall, cancer risk among children with congenital anomalies is highest in those under five years of age. For children with non-chromosomal anomalies, the greatest risk of any cancer is observed during the first year of life, whereas for those with chromosomal anomalies, the increased risk is most pronounced between ages one and four [39]. Importantly, the elevated cancer risk persists into adulthood for both anomaly types [40]. Von Behren et al. further reported that this persistence into older ages was evident only for chromosomal anomalies [41].

Most studies have found a positive association between childhood cancer and congenital anomalies. However, variations in the magnitude of risk and in the specific anomaly–cancer associations have been reported. Children with any congenital anomaly have been shown to have a 1.5- to 4.5-fold increased risk of childhood cancer overall [24,42], and a 2.3- to 16.7-fold increased risk among those with chromosomal anomalies [14,18].

Some discrepancies have emerged in studies focusing on specific childhood cancer cancers, such as neuroblastoma. Bjørge et al. [27] reported a 2.3-fold increased risk of neuroblastoma in children with congenital anomalies diagnosed before 18 months of age. Chow et al. [28] and Menegaux et al. [29] reported 6.9- and 7.5-fold risks, respectively, in children with major congenital anomalies. Rios et al. [30] found a 3.6-fold increased risk in children under 18 months with at least one major anomaly. Differences in study design, population characteristics, data sources, case definitions, and analytical methods may partially explain these inconsistencies [3].

Although the association between childhood cancers and congenital anomalies has been extensively documented, few studies have evaluated cancer survival in children with congenital anomalies [43,44,45]. A better understanding of survival outcomes in this population could enhance clinical management and survivorship care.

One of the main challenges in identifying associations between childhood cancers and congenital anomalies is the limited population size, given the rarity of both conditions. Population-based cancer and congenital anomaly registries have proven to be valuable resources for conducting such epidemiological studies [14,17,18,19,20,21,25].

The European Cancer Information System (ECIS) and the European network of population-based registries for the epidemiological surveillance of congenital anomalies (EUROCAT) collect and report data on childhood cancers and major congenital anomalies, respectively, at the population level.

ECIS, developed by the Joint Research Centre of the European Commission, is a comprehensive health and research infrastructure that harmonizes data from over 100 population-based cancer registries across Europe. It supports cancer burden monitoring, health policy development, and research on prevention, treatment, and care. Currently, ECIS provides childhood cancer data from 96 registries across 30 countries [46].

EUROCAT, in turn, fosters the development of registries that collect standardized and comparable data on major congenital anomalies diagnosed during the first year of life. Its primary objectives include identifying teratogenic exposures and evaluating the impact of prevention and prenatal screening policies. EUROCAT data are contributed by 32 registries across 18 European countries, all adhering to a standardized methodology [47,48].

Both ECIS and EUROCAT follow European and international standards, ensuring data comparability across regions. Together, they offer a robust and harmonized dataset for analyzing cancer risk and survival outcomes in children with congenital anomalies.

In the Valencian Region (VR), two high-quality population-based registries—the Childhood and Adolescent Population-Based Cancer Registry (CAPBCR-VR), which contributes to ECIS, and the Congenital Anomalies Population-Based Registry (CAPBR-VR), a full member of EUROCAT [49,50] provide a unique opportunity to evaluate cancer risk and survival in children with congenital anomalies. This study may serve as a pilot and feasibility model for other European regions with access to both types of registries.

The aims of this study were: (1) to estimate the cancer risk in children under 15 years of age diagnosed with at least one major congenital anomaly during the first year of life, born between 2007 and 2020 and residing in the VR; and (2) to assess survival differences across three groups: children with both cancer and congenital anomalies, children with cancer only, and children with congenital anomalies only.

## 2. Materials and Methods

The VR, located along Spain’s eastern Mediterranean coast, had a population of 708,167 individuals less than 15 years of age and an average of approximately 45,000 births per year during the study period (2007–2020) [51,52].

### 2.1. Data Sources and Case Selection

The CAPBCR-CV provided the childhood cancers diagnosed during the period 2007–2020, born between 2007 and 2020 and residing in the VR. Children with at least one major congenital anomaly, diagnosed during the first year of life, born in 2007–2020 and residing in the VR were obtained from the CAPBR-CV. These two datasets were linked by authorized personnel using a unique identification number to identify children diagnosed with both childhood cancer and congenital anomalies. All analyses were conducted using anonymized data, ensuring that no individual included in the study could be identified.

Children were categorized into three groups: (1) children with both childhood cancer and congenital anomalies (CC + CA group); (2) children with childhood cancer only (CC group) and; (3) children with congenital anomalies only (CA group).

Childhood cancers were classified according to the International Classification of Childhood Cancer, 3rd edition, 2017 update (ICCC-3-2017), including both main groups and subgroups [53].

Major congenital anomalies were defined and grouped following the EUROCAT Guide 1.5 [54]. In addition, specific analyses were performed for non-genetic anomalies, defined as all anomalies not classified as genetic disorders according to EUROCAT. Within the group of genetic disorders, two subgroups were considered: chromosomal anomalies and other genetic disorders. Only Down syndrome was included in the analysis, as it was the sole chromosomal anomaly diagnosed among children with cancer.

All children with cancer and/or congenital anomalies were followed until 31 December 2024. Vital status, date of death, and date of loss to follow-up were obtained using the Mortality Registry of the VR, the Spanish National Death Index, and the Population Information System of the VR.

### 2.2. Statistical Analysis

Standardized incidence ratios (SIRs) and corresponding 95% confidence intervals (95% CI) were computed to estimate the cancer risk among children with congenital anomalies, using CAPBCR-VR rates as the reference. Each child contributed person-years of observation from birth until the earliest of the following events: cancer diagnosis, departure from the VR, death, or 31 December 2020.

Analyses were conducted for all congenital anomalies combined, as well as separately for each EUROCAT-defined anomaly group with at least five cases in the CC + CA group.

Kaplan–Meier survival analysis was used to estimate survival functions, and the log-rank test was applied to compare survival curves.

First, overall survival was estimated and compared between the CC + CA and CA groups. Overall survival was defined as the time from birth to death, departure from the VR, or end of follow-up (31 December 2024), whichever occurred first. One-, three-, and five-year survival rates with 95% CIs were calculated for each group.

Second, overall survival was estimated and compared between the CC + CA and CC groups. In this analysis, overall survival was defined as the time from cancer diagnosis to death, departure from the VR, or end of follow-up (31 December 2024), whichever occurred first. One-, three-, and five-year survival rates with 95% CIs were also calculated for each group.

## 3. Results

The CAPBCR-VR recorded 911 childhood cancer cases (in 909 children) diagnosed between 2007 and 2020, all born during the same period. A total of 11,742 live births with major congenital anomalies were registered between 2007 and 2020. Among these, 66 children were identified as having both childhood cancer and congenital anomalies.

**Figure 1:**
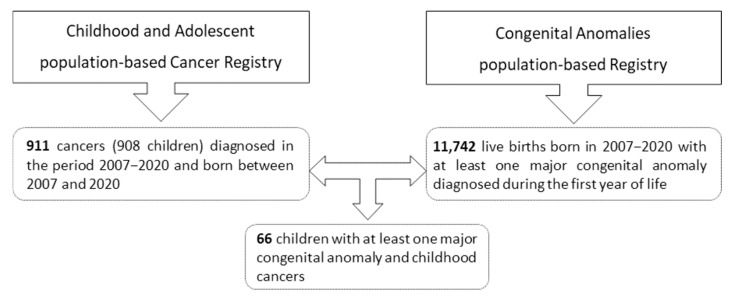
Number of cases provided by the population-based registries of the Valencian Region.

Thus, 7.2% of children with childhood cancer had at least one major congenital anomaly, while approximately 0.6% of children with major congenital anomalies were diagnosed with childhood cancer. Figure 1 presents the number of cases reported by the CAPBCR-VR and CAPBR-VR.

The average number of major congenital anomalies per child was 1.6 in the group of congenital anomalies only (CA group) and 1.8 in the group with both childhood cancer and congenital anomalies (CC + CA group). The proportion of children with more than one major congenital anomaly was significantly higher in the CC + CA group (52.2%) compared to the CA group (34.3%).

**Figure 2:**
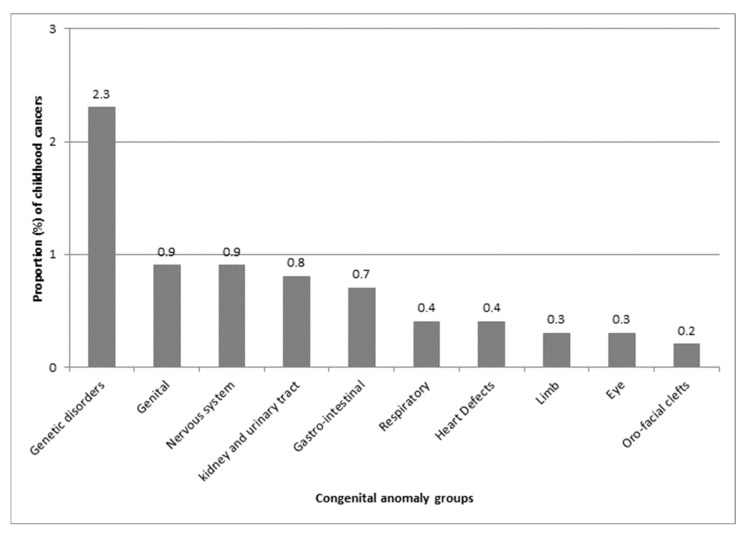
Proportion (%) of childhood cancer by congenital anomaly group, as defined by EUROCAT (European network of population-based registries for the epidemiological surveillance of congenital anomalies).

The highest proportions of childhood cancer were observed in children with genetic disorder anomalies (2.3%), followed by genital anomalies (0.9%), nervous system anomalies (0.9%), and kidney and urinary tract anomalies (0.8%) (Figure 2).

**Figure 3:**
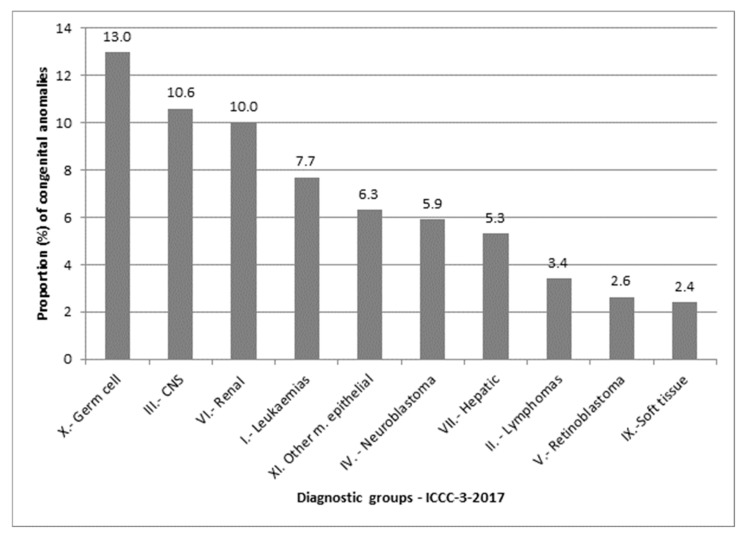
Proportion (%) of congenital anomalies by cancer diagnostic group, based on the International Classification of Childhood Cancer, 3rd edition (ICCC-3), 2017 update.

The proportions of congenital anomalies among children with specific cancer types were as follows: 13.0% in those with germ cell malignant tumours (ICCC-3-2017 diagnostic group X), 10.6% in central nervous system tumours (group III), and 10.0% in renal malignant tumours (group VI). No major congenital anomalies were found in children with malignant bone tumours (group VIII) or other and unspecified malignant neoplasms (group XII) (Figure 3).

Four children with congenital anomalies were diagnosed with cancer at birth: two with neuroblastoma, one with malignant teratoma, and one with desmoplastic infantile astrocytoma. Neonatal cancers (diagnosed within the first 28 days of life) were observed in 10 children (15.2%) with congenital anomalies and in 15 children (1.8%) without congenital anomalies. Cancer was diagnosed before the first year of life in 39.4% of children with congenital anomalies, and in 87.9% before the age of five. Among children without congenital anomalies, these proportions were 17.3% and 64.8%, respectively.

The most frequent cancer types in children with congenital anomalies were central nervous system tumours (36.4%) and leukaemias (33.3%). Among children with genetic disorder anomalies, central nervous system tumours and leukaemias each accounted for 42.3% of cases. Of the eight cancers diagnosed in children with nervous system anomalies, six (75%) were central nervous system tumours. In children with Down syndrome, leukaemia accounted for 88.9% of all cancer diagnoses.

An increased risk of cancer was observed in children with congenital anomalies compared to the reference population (SIR = 5.5; 95% CI: 4.3–7.0). This risk was markedly higher in children with genetic disorders (SIR = 26.6; 95% CI: 17.3–37.8) than in those with non-genetic anomalies (SIR = 3.7; 95% CI: 2.6–4.9). Table 1 presents SIRs and their corresponding 95% confidence intervals for specific congenital anomaly groups. The subgroup of genetic anomalies excluding chromosomal abnormalities showed the highest risk of childhood cancer (SIR = 37.8; 95% CI: 21.9–57.8). Children with Down syndrome had a 22-fold increased risk of developing childhood cancer compared to the reference population.

**Figure 4:**
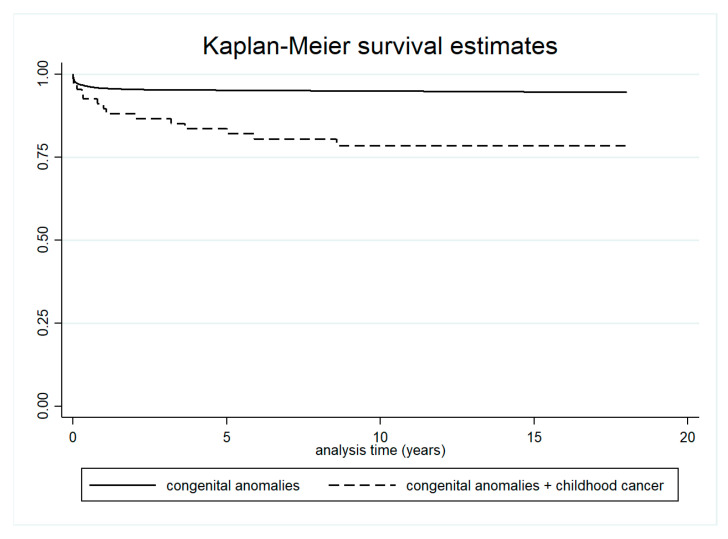
Survival in children with both cancer and congenital anomalies, and in children with congenital anomalies only.

**Figure 5:**
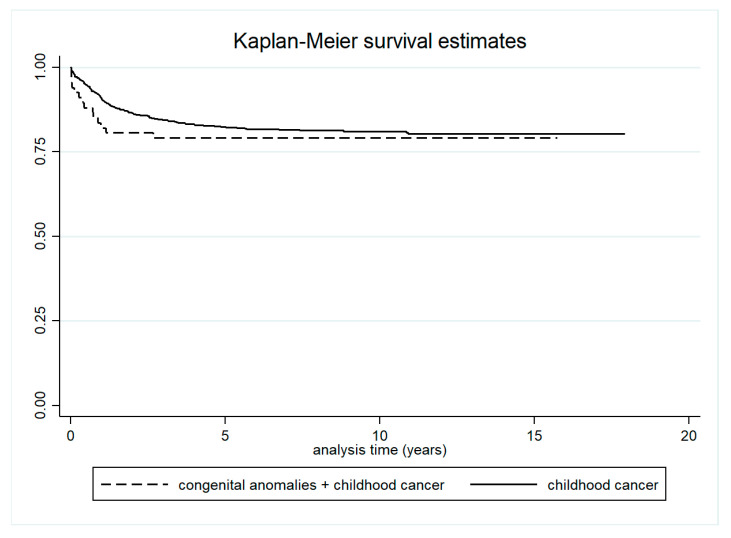
Survival in children with both cancer and congenital anomalies, and in children with cancer only.

Five-year survival was significantly lower in children with both cancer and congenital anomalies (83.6%; 95% CI: 72.3–90.6) compared to children with congenital anomalies only (95.2%; 95% CI: 94.8–95.5) (Table 2 and Figure 4). Survival was also lower in children with both conditions compared to those with cancer only, although this difference was not statistically significant (Table 3 and Figure 5).

## 4. Discussion

The present study investigated the risk of childhood cancer among children diagnosed with at least one major congenital anomaly during the first year of life, as well as the impact of these anomalies on survival. Data were obtained from two population-based registries established in the VR, the CAPBCR-VR and the CAPBR-VR.

Although several population-based studies examining the association between childhood cancer and congenital anomalies have been conducted in other countries, this is the first analysis of its kind carried out in Spain. To date, only one study based on historical series of neonatal tumours from a Spanish hospital has reported the frequency of neonatal tumours associated with congenital anomalies [55].

During the study period, major congenital anomalies were identified in approximately 7% of children diagnosed with cancer—lower than the proportions reported in some studies [19,20], but higher than the 0.2% reported by Collins et al. [26]. Berbel et al. [55] found that 21% of children with congenital anomalies developed cancer within the first 28 days of life. In our cohort, this proportion was lower, at 15%. Variations in the definition and classification of congenital anomalies may partially explain these differences.

In the current population-based study, children with at least one major congenital anomaly had an approximately 5-fold increased risk of developing childhood cancer compared to the reference population. Although the association between childhood cancer and congenital anomalies has been reported by several authors [14,15,16,17,18,21,22,24,25], the magnitude of risk observed in those studies was generally lower, ranging from 1.5 to 4.5. Differences in study period, data sources, and methodology may partly explain these discrepancies.

The highest cancer risks were observed among children with genetic disorders, excluding chromosomal anomalies, and those with Down syndrome, with 38-fold and 22-fold increased risks, respectively, compared to the general population. Altman et al. reported similar cancer risks for children with Down syndrome [14]. Bjørge et al. found even higher risks in Norway and Sweden [15], and Mili et al. reported comparable findings in Atlanta and Iowa [21,22]. In our study, leukaemia was the most frequently diagnosed cancer among children with Down syndrome, consistent with previous reports [14,15,21,22].

We were unable to directly compare the 26-fold increased cancer risk observed in children with genetic disorder anomalies to findings from other studies. The EUROCAT-defined group encompasses genetic syndromes, hereditary skin disorders, skeletal dysplasias, and chromosomal anomalies. Although some researchers have analyzed chromosomal and non-chromosomal anomalies separately [16,20,24], these classifications are not directly comparable to those used in our analysis. Due to the small number of cases with both congenital anomalies and childhood cancer, Down syndrome was the only chromosomal anomaly diagnosed among affected children in our cohort. The cancer risk in this subgroup was 22 times higher than in the reference population, surpassing the 10- to 14-fold increase reported in previous studies [16,20,24].

There is growing evidence that inherited genetic factors play a substantial role in the development of childhood cancer, and associations have also been reported between non-chromosomal anomalies and increased cancer susceptibility. It is estimated that approximately 10% of children with cancer have an underlying cancer predisposition syndrome. Understanding the etiology of childhood cancer and the associated syndromes is essential for genetic counseling, therapeutic decision-making, and the development of effective surveillance programs [7,8,9,10,56,57].

Most cancers in children with congenital anomalies of the nervous system were central nervous system (CNS) tumours (75%). However, we did not identify any other non-genetic disorder anomalies that were specifically associated with cancers occurring in the same organ. The association between nervous system anomalies and CNS tumours has also been reported by other authors [14,15,24,42].

We observed a statistically significant reduction in overall survival among children with both childhood cancer and congenital anomalies, compared to those with congenital anomalies alone. A lower—but not statistically significant—survival was also found when comparing children with both conditions to those with childhood cancer only, particularly within the first year after cancer diagnosis. Janitz et al. similarly reported no significant differences in cancer survival between children with and without congenital anomalies [43].

In our cohort, the proportion of children with more than one major congenital anomaly was significantly higher in children with both conditions compared to children with congenital anomalies only. This increased comorbidity may partially explain the poorer survival observed in children with both childhood cancer and congenital anomalies. Differences in the distribution of tumour types and congenital anomalies may also impact survival outcomes.

Divergent findings have been reported for specific combinations of congenital anomalies and cancer types. For example, children with acute myeloid leukaemia and congenital anomalies (excluding Down syndrome) showed poorer survival compared to children without anomalies or with Down syndrome. In contrast, similar survival rates were observed across these three groups for acute lymphoblastic leukaemia [44].

Certain types of congenital anomalies may influence treatment protocols. For instance, children with congenital heart anomalies and lymphoma may receive reduced doses or alternative therapies due to the cardiotoxic effects of standard chemotherapeutic agents, potentially resulting in less effective treatment [45].

Studies evaluating cancer survival in children with congenital anomalies remain scarce. More consistent and comprehensive evidence is needed to support planning and improve health care for this vulnerable population.

One of the strengths of our study is the use of data from two well-established, high-quality population-based registries, both adhering to international and European standards and classifications. This ensures that our findings are comparable to those reported in other European studies using similar data sources.

To our knowledge, this is the first population-based study conducted in Spain to assess the increased cancer risk in children with congenital anomalies. It is also one of the few studies to evaluate survival outcomes in children with both childhood cancer and congenital anomalies, compared to those with only one of these conditions.

The main limitation of our study relates to the rarity of both childhood cancer and congenital anomalies. Although the study cohort included 11,742 live births with at least one major congenital anomaly and 908 children diagnosed with cancer, only 66 children presented with both conditions. This limited our ability to conduct more detailed analyses by specific anomaly types and cancer subgroups due to small sample sizes. However, we were able to compare cancer risk between children with and without genetic disorders. Furthermore, we divided the genetic disorder group into two subgroups: those with genetic disorders excluding chromosomal anomalies, and those with Down syndrome—the only chromosomal anomaly diagnosed among children with cancer in our cohort.

Nevertheless, our findings are consistent with those reported in previous studies, and survival estimates were obtained for all three groups: children with childhood cancer and congenital anomalies, children with childhood cancer only, and children with congenital anomalies only.

Since the implementation of the General Data Protection Regulation [58] does not allow centralized data linkage, a collaborative effort involving other European regions with population-based cancer registries (contributing to ECIS) and congenital anomaly registries (EUROCAT full members) would facilitate more detailed analyses and improve data comparability across Europe.

In conclusion, we observed an increased risk of cancer in children with congenital anomalies. The highest risks were identified among those with genetic disorders, particularly Down syndrome. Cancer tended to be diagnosed at an earlier age in children with congenital anomalies, and overall survival was poorer in those affected by both cancer and congenital anomalies compared to children with only one of these conditions. These findings highlight the need for targeted oncological surveillance following the diagnosis of a congenital anomaly, especially in children with genetic disorders, who represent an extremely high-risk population.

## Figures and Tables

**Table 1:** Standardized incidence ratios and 95% confidence intervals by congenital anomaly group, as defined by EUROCAT (European network of population-based registries for the epidemiological surveillance of congenital anomalies).

Congenital Anomaly Group	* N. of Children with Congenital Anomalies	N. of Children with Childhood Cancers and Congenital Anomalies (%)	^∞^ SIR ^Ω^ (95% CI)
All genetic disorders	1103	26 (2.3%)	26.6 (17.3–37.8)
– Genetic disorders, excluding chromosomal anomalies	534	17 (3.2%)	37.8 (21.9–57.8)
– Down syndrome	395	9 (2.2%)	22.2 (10.1–39.1)
Non-genetic disorder	10,572	40 (0.4%)	3.7 (2.6–4.9)
Nervous system	908	8 (0.9%)	9.9 (4.2–18.0)
Genital	847	8 (0.9%)	9.1 (3.9–16.5)
Kidney and urinary tract	1817	15 (0.8%)	8.3 (4.7–13.1)
Gastrointestinal	933	7 (0.7%)	7.9 (3.1–14.8)
Heart Defects	4951	19 (0.4%)	3.8 (2.3–5.7)

* N: Number; ^∞^ SIR: Standardized incidence ratio; ^Ω^ (95% CI): 95% confidence intervals.

**Table 2:** Survival in children with both congenital anomalies and cancer, and in children with congenital anomalies only.

	Survival in Children with Cancers and Congenital Anomalies	Survival in Children with Congenital Anomalies
1 year	89.6% (79.3–94.9)	95.8% (95.4–69.1)
3 years	86.6% (75.8–92.8)	95.3% (95.0–95.9)
5 years	83.6% (72.3–90.6)	95.2% (94.8–95.5)

**Table 3:** Survival in children with both congenital anomalies and cancer, and in children with cancer only.

	Survival in Children with Cancers and Congenital Anomalies	Survival in Children with Cancers
1 year	82.1% (70.6–89.4)	90.6% (88.9–92.4)
3 years	79.1% (67.3–87.1)	84.5% (81.9–86.8)
5 years	79.1% (67.3–87.1)	82.3% (79.3–84.7)

## Data Availability

The datasets presented in this article are not available because a specific request is necessary. Requests to access the datasets should be directed to the Public Health Directorate of the Generalitat Valenciana through their website (https://www.san.gva.es/ca/web/salut-publica/solicitud-de-informacion), (accessed on 31 October 2025).

## Abbreviations

The following abbreviations were used in this manuscript:

CA	Congenital anomalies
CC	Childhood cancers
CC + CA	Childhood cancer and congenital anomalies
CAPBR-VR	Congenital Anomalies population-based Registry of Valencian Region
CAPBCR-VR	Childhood and Adolescent population-based Cancer Registry of Valencian Region
ECIS	European Cancer Information System
EUROCAT	European network of population-based registries for the epidemiological surveillance of congenital anomalies
CAPBCR-VR	Childhood and Adolescent population-based Cancer Registry
ICCC-3-2017	International Classification of Childhood Cancer, 3rd edition, update of 2017
SIR	Standardized incidence ratio
95% CI	95% Confidence intervals
VR	Valencian Region
